# The effect of two iso-caloric meals containing equal amounts of fats with a different fat composition on the inflammatory and metabolic markers in apparently healthy volunteers

**DOI:** 10.1186/1476-9255-10-3

**Published:** 2013-01-31

**Authors:** Olga Raz, Arie Steinvil, Shlomo Berliner, Tovit Rosenzweig, Dan Justo, Itzhak Shapira

**Affiliations:** 1 The Departments of Diet and Nutrition, The Tel-Aviv Sourasky Medical Center, 6 Weizman St, Tel Aviv, Israel; 2 Cardiology, Tel Aviv University, Tel Aviv, Israel; 3 Internal Medicine “D” & “E” the Tel Aviv Sourasky Medical Center affiliated to the Sackler Faculty of Medicine, Tel Aviv University, Tel Aviv, Israel; 4 The Department of Molecular Biology at the Faculty of Natural Sciences, the Ariel University of Samaria, Samaria, Israel; 5 Department on Nutritional Sciences, the Ariel University of Samaria, Israel

**Keywords:** Inflammation, Monounsaturated fats, Saturated fat, Nutrition

## Abstract

**Background:**

Little is known about the time-course of the postprandial appearance of macronutrient-induced inflammatory response. Our aim was to investigate the postprandial inflammatory and metabolic response following high fat, high caloric popular meals in apparently healthy participants.

**Methods:**

Fifty four apparently healthy normal weight volunteers (BMI of 25.9±0.9) were given two iso-caloric meals with similar amounts but different composition of fats: a meal high in monounsaturated fats (MUFA), and a meal high in saturated fat (SFA). Three main effects and the interactions between them were analyzed: the time (before and 2 and 4 hours following the meals), the meal (MUFA or SFA) and the gender.

**Results:**

The effect of time from the meal on hs-CRP level was highly significant (*p*=0.004). The highest responses were observed 2 hours after the meal (*p*=0.002). A statistically significant interaction was found between the time and the meal (*p*≤0.0001), which reflects the higher increase in hs-CRP values 2 hours after the SFA meal, with no effect by the MUFA meal. The white blood cell counts were affected significantly by the time (*p*≤0.0001) however, other inflammatory markers (fibrinogen, IL-6, TNFα, ICAM and VICAM) were not. All the metabolic markers (insulin, glucose, HOMA-R, QUICKI and triglycerides) were affected by the time (*p*≤0.0001), with no interactions observed.

**Conclusions:**

Metabolic and modest inflammatory changes occur within a few hours after the ingestion of a high SFA meal in apparently healthy adults.

## Background

Understanding the nutrients and foods that are likely to promote cardiac health has grown substantially in the past decades owing to knowledge achieved on the molecular mechanisms of atherosclerosis and the metabolic effects of various nutrients and foods. Previous studies have shown that a ‘Western type', energy dense, refined diet, may lead to the development of a positive energy balance, weight gain, obesity, particularly visceral obesity and eventually to be a key promoter of low-grade systemic inflammation [1-3] and metabolic syndrome abnormalities [4,5]. Various diets have been explored with reference to their capability for improving body weight and components of the metabolic syndrome as well as the intensity of low-grade internal inflammation as part of the atherothrombotic disease [6-10].

Little is known about the time-course of postprandial triglyceride serum levels and their relation to the appearance of a macronutrient-induced inflammatory response. Their importance may lie in the fact that an early postprandial inflammatory response might precede a potentially hazardous transient state of insulin resistance and hypertriglyceridemia [11]. In fact, mediators of inflammation have been clearly shown to be involved in the induction of an insulin resistant state [12,13]. A transient, macronutrient-related, microinflammatory response could explain, at least in part, the appearance of a dynamic insulin resistant state which could be responsible for a postprandial hypertriglyceridemic response [14-23]. In the present study we have investigated the metabolic and inflammatory impact of the fat composition of a single meal, rather than its fat amount, has any effect on selected metabolic and inflammation-sensitive biomarkers which are involved in the atherosclerosis process.

## Methods

### Study population

Sixty staff members (physicians and nurses) of the Tel-Aviv Sourasky Medical Center volunteered to take part in the study and signed a written consent according to the instructions of the Institutional Ethics Committee. Included were apparently healthy non obese (BMI 25.9 kg/m^2^) subjects with no recent acute or chronic inflammatory disease, not using anti inflammatory and immunosuppressive drugs and steroids. Six potential subjects were excluded: two subjects suffered from a recent upper respiratory tract infection, two used non-steroidal anti-inflammatory drugs on a regular basis for back pain, one was taking statins for hypercholesterolemia, and one had acute tooth pain. The decision to choose healthy subjects was made in order to determine whether a single food has a pro or anti inflammatory effect on the general population.

### Study design

The chosen meals represented two very popular meals habitually preferred by the general population: 1. Chicken sausages with fried potatoes, ketchup and mayonnaise (defined as SFA); 2. Pasta with olive oil, ketchup and nuts (defined as MUFA). Both meals are consumed by adults and children on an almost daily basis. Both meals are high in calories and fat. The composition of the meals used in the study was close to that of the meals eaten at home or in fast-food restaurants.

All participants ate two iso-caloric meals with similar amounts of fat. The participants were randomly divided into two groups. One group began with the meal that was high in saturated fats (SFA), and continued consuming the meal high in mono-unsaturated fats (MUFA) after three weeks. The second group began with the high complex high MUFA meal, and began consuming the high SFA after three weeks. Blood samples were taken following a 12-hour overnight fast, after which one of the meals was provided as breakfast. The second and third blood samples were taken two and four hours after the meal. Plasma lipids, glucose, insulin, erythrocyte sedimentation rate (ESR), fibrinogen, white blood cell count (WBCC), and high-sensitive C-reactive protein (hs-CRP) were determined. The insulin resistance index was calculated according to the Homeostatic Model Assessment for Insulin Resistance (HOMA-R) [24] and the Quantitative Insulin Sensitivity Check Index (QUICKI) [25,26].

### Meals composition

High saturated fat (SFA) meal included 1162 kilocalories and 57% fat, of which 24 grams were saturated fatty acids, 33 grams – mono-unsaturated fatty acids, and 17 grams – poly-unsaturated fatty acids. High mono-unsaturated fat (MUFA) meal included 1161 kilocalories, 56% fat, of which 8 grams were saturated fatty acids, 51 grams - mono unsaturated fatty acids, and 14 grams – poly-unsaturated fatty acids. The fat content of both meals was adjusted to the body surface area (BSA) of each participant which was calculated according to the formula: BSA (m^2^) = square root of the height (cm) x the weight (kg)/3600 [27,28].

The data were calculated using the nutrients computer program of the Israeli Ministry of Health - “Tzameret”, which is based on the Israeli Food Composition Tables [29].

### Laboratory methods

All biochemical assessments were carried out by the Sourasky Medical Center Laboratory, using the same standard laboratory methods. The laboratory has an ISO 9001:2000 standard certification and thus implements and maintains the quality management system, as required.

Venous blood was drawn at 8 A.M. following a 12-hour overnight fast, and 2 and 4 hours following the meal. The WBCC was estimated using a Coulter STKS electronic analyzer (Beckman Coulter, Nyon, Switzerland), the ESR using the method of Westergren, quantitative fibrinogen using the method of Clauss [30] with a Sysmex 6000 autoanalyzer (Sysmex Corporation, Hyaga, Japan) and the hs-CRP was determined with a Boehring BN II Nephelometer (DADE Boehring, Marburg, Germany) using the method of Rifai et al. [31]. Routine biochemistry, including the blood glucose and lipid profile, was performed using the Roche/Hitachi 747 Analyzer (Roche Diagnostics, Mannheim, Germany). Insulin levels were determined using the INSIK-5 (CIS, Grif sur Yvette, France) radioimmunoassay kit.

Measurement of cytokines and adhesion molecules was performed in the Laboratory of Cell Physiology of the Ariel University Center (Ariel 40700, Israel), by its director, Dr. Tovit Rosenzweig. The concentration of cytokines (IL-6 and TNF-α) and adhesion molecules (sICAM1 and sVCAM1) was measured within 6-12 months in serum samples that were stored at -80°C immediately after collection. Measurements were performed using high sensitive quantitative sandwich enzyme immunoassay (Quantikine HS Human TNFα Immunoassay, Quantikine HS Human IL-6 Immunoassay, Quantikine human soluble ICAM-1/CD54 Immunoassay, and Quantikine human soluble VCAM-1 Immunoassay, (R&D Systems, Minneapolis, USA) according to the manufacturer's instructions. Samples were pipetted into specific monoclonal antibody-precoated wells, and any IL6 or TNF-α or VCAM-1 or ICAM-1 present was sandwiched by the immobilized antibody. After washing away any unbound substances, an enzyme-linked polyclonal antibody specific for the detected cytokine or adhesion molecule was added to the wells (alkaline phosphatase for IL6, TNFα; horseradish peroxidase for ICAM1, VCAM1). The wells were washed to remove any unbound antibody-enzyme reagent, and a substrate solution was added. After an incubation period, an amplifier solution was added to the wells and color developed in proportion to the amount of the cytokine bound in the initial step. Color development was stopped by addition of stop solution (sulfuric acid) and the intensity of the color was measured using a Tecan Infinite F200 fluorescence microplate reader (Tecan, Salzburg, Austria) at a test wavelength of 490 nm and a reference wavelength of 650 nm for cytokines, or at a test wavelength of 450 nm and reference wavelength of 540 nm or 650 nm for sVCAM1 or sICAM1, respectively.

Peripheral insulin resistance and sensitivity were estimated using the Homeostasis Model Assessment (HOMA-R) calculated as glucose (mg/dl) x insulin (μU/ml)/405 [24] and the Quantitative Insulin Sensitivity Check Index (QUICKI) calculated as 1/(log insulin + log glucose [25,26].

Anthropometric measurements: Height and weight were measured without shoes. The subjects were weighed on electronic scales, without shoes and in light clothing. The precision of the scale was to 100 grams. Hip and waist circumferences were measured twice with a tape measure by the same investigator in order to avoid interpersonal differences. If the two measurements differed by more than 0.5 cm, a third measurement was taken. If two measurements were similar – the mean was calculated, if not – the mean was calculated with the 3^rd^ measurement and with the one closest to it. Body mass index was calculated using the equation: BMI=Weight (kg)/Height^2^ (m).

### Statistical analysis

All data were summarized and displayed as means ± standard errors for the continuous variables, such as age, body mass index (BMI), inflammation-sensitive biomarkers, etc., using the descriptive procedure. Because the hs-CRP level had a non-normal distribution, a logarithmic transformation was used to convert it to a normal distribution for all statistical procedures (i.e., the *t*-test, multiple factors analysis and correlations). All hs-CRP results are therefore presented as a back-transformed geometrical mean and standard error.

Repeated measures ANOVA with multiple factors was performed for all continuous variables in order to compare the repeated measures at different times after the same meal and between the two meals, as well as to establish interactions between the repeated measures and multiple factors (gender and insulin level - treatment groups).

A paired sample two-tailed Student's *t*-test analysis was performed in order to compare the characteristics of responders and non responders.

Pearson correlation coefficients were used for confounding variables in order to study the associations between postprandial hs-CRP serum levels and the postprandial lipid, glucose and insulin serum levels. These correlations were studied for the whole study population. The significance level used for all of the above analyses was *p*<0.05. All statistical evaluations were carried out using the SPSS package (SSPS Inc., Chicago, IL, USA).

## Results

The study included 54 participants (38 females, 16 males), who served as controls of themselves, with a mean age of 41.7±3.1 years and a mean BMI of 25.9±0.9. Other details are provided in the Methods section (pp. 20-24). The main effects that were analyzed included: the meal (SFA or MUFA), the time (before and 2 and 4 hours following each meal), and the gender of the participants. First, we analyzed the effects of the two meals on the inflammatory markers within the treatment groups. These results are summarized in Table 1.

**Table 1:** SFA and MUFA meals: comparison of the inflammatory markers among treatment groups, mean ± SEM

**Treating groups meals**		**CRP (mg/l)**	**ESR (mm/hr)**	**Fibrinogen (mg/dl)**	**WBC x10**^**9**^**/L**	**IL6 (pg/ml)**	**TNFά (pg/ml)**	**VCAM (ng/ml)**	**ICAM (ng/ml)**
SFA: Women	0h	2.77±0.5	16.8±1.9	280.5±14	7.2±0.3	2.16±0.3	3.08±0.3	419.7±27.1	211.9±17.4
	2h	2.94±0.5	17.7±1.9	285.2±11.9	7.8±0.3	2.54±0.6	2.64±0.2	404.5±23.0	209.3±16.6
	4h	2.76±0.5	18.9±1.8	279.7±12.3	8.2±0.3	2.56±0.3	2.89±0.3	405.6±24.8	204.9±17.1
MUFA: Women	0h	2.82±0.5	18.7±1.7	297.0±11.4	7.4±0.3	2.13±0.2	3.12±0.3	421.9±22.7	220.6±19.2
	2h	2.73±0.5	18.3±1.8	288.4±11.0	7.9±0.3	2.09±0.2	3.33±0.3	410.6±23.5	216.1±19.9
	4h	2.72±0.5	18.1±1.9	288.4±11.1	8.1±0.3	2.46±0.3	2.85±0.3	435.5±25.9	231.0±20.0
SFA: Men	0h	2.3±0.5	8.5±0.9	261.9±15.4	6.6±0.4	2.29±0.6	2.96±0.4	382.3±32.9	200.8±22.9
	2h	2.85±0.6	9.2±1.0	270.2±17.4	7.3±0.4	2.15±0.5	2.97±0.4	388.1±32.1	196.6±18.6
	4h	2.72±0.7	9.9±1.0	258.7±10.7	7.6±0.3	2.28±0.4	2.9±0.4	387.5±29.5	193.6±18.6
MUFA: Men	0h	2.26±0.6	11.5±3.3	266.9±11.7	7.2±0.4	4.07±1.4	2.44±3.6	397.2±35.1	197.0±16.0
	2h	2.27±0.6	11.6±3.1	262.4±11.4	7.3±0.4	2.24±0.5	2.9±0.4	397.4±33.4	191.2±15.6
	4h	2.3±0.6	10.1±1.6	249.9±20.3	7.3±0.4	3.16±0.6	2.79±0.5	395.4±31.7	188.7±12.6
**Main effects**									
Meal		0.4	0.2	0.7	0.6	0.3	0.9	0.5	0.6
Time		**0.004**	0.5	0.1	**<0.0001**	0.3	0.9	0.5	*0.06*
0-2		**0.002**			**<0.0001**				
2-4		0.1			**0.01**				
0-4		0.3			**<0.0001**				
Gender		0.7	**0.007**	0.2	0.2	0.5	0.7	0.5	0.4
**Interaction**									
Meal x Time		**<0.0001**	**0.03**	0.3	*0.06*	0.3	0.2	0.7	0.6
0-2		**<0.0001**	0.1						
2-4		**0.04**	**0.04**						
0-4		**0.05**	**0.007**						
Meal x Gender		0.6	0.5	0.3	0.9	0.1	0.3	0.9	0.3
Time x Gender		**0.05**	0.6	0.6	0.3	0.1	0.5	0.2	0.7
Meal x Time x Gender		0.4	0.7	0.9	0.2	0.5	0.6	0.5	0.6

WBCC, white blood count; ESR, erythrocyte sedimentation rate; hs-CRP, C-reactive protein**;** SFA, high saturated fat meal; MUFA, high monounsaturated fat meal.

As shown, the main effect of the time on the hs-CRP level was statistically significant (*p*=0.004), albeit moderate. The highest responses were observed 2 hours after the meal (*p*=0.002). A statistically significant interaction was found between the time and the meal (*p*≤0.0001), which reflects the higher increase in hs-CRP values 2 hours after the SFA meal, with no effect by the MUFA meal. There was a significant interaction (*p*=0.05) between the time and the gender. The increase in the hs-CRP level after 2 hours following the SFA meal was higher in the men than in the women: 23.9% vs 6%. In the women, the hs-CRP level after the SFA meal returned to normal 4 hours postprandially, whereas it remained high in the men (18.2% higher than before the meal).

In contradistinction to the hs-CRP, the main effect of gender was significant in the results of the ESR (*p*=0.007), since the values were much higher in the women than in the men. There was also a statistically significant interaction between the time and the meal (*p*=0.03), particularly after 2 and 4 hours (*p*=0.04 and 0.007, respectively), with no dependence on the gender. As shown, there was a continuous increase in ESR values after the SFA meal in both the women and the men (5.3% after 2 hours and 12.5% after 4 hours for the women, 8.2% after 2 hours and 16.5% after 4 hours for the men) with no increase after the MUFA meal.

As shown in Table 1, WBCC values were affected significantly by the time (*p*≤0.0001) and increased after both meals. The interaction between the time and the meal was statistically borderline (*p*=0.06): WBCC values increased by 13.5% after 4 hours following the SFA meal compared with a relatively lower decrease of 6.4% after the MUFA meal (women and men pooled). The other inflammatory markers (fibrinogen, IL-6, TNFα, ICAM and VICAM) were not affected by the meals and did not change after the meals.

**Figure 1:**
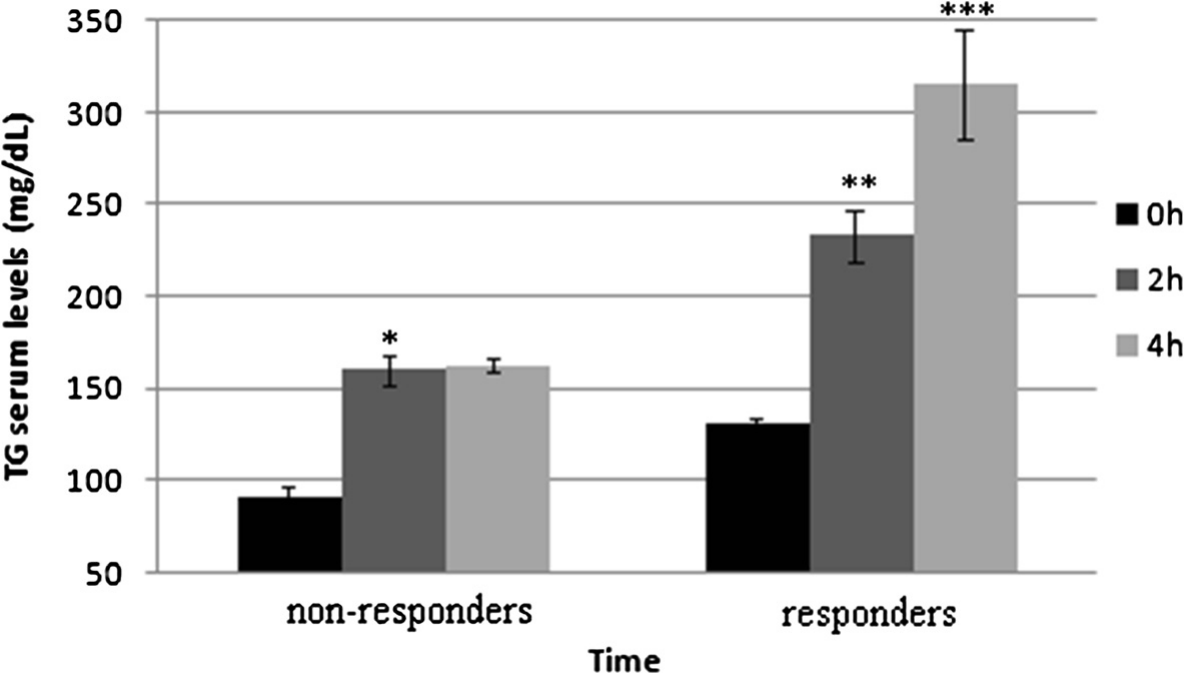
Interrelations between triglyceride levels in responders and non-responders in the fasting state and 2 and 4 hours postprandially, mean ± SEM.

Since the increase in hs-CRP levels after the SFA meal was observed only in some of the participants, the participants were divided into responders (i.e., with at least 20% elevation of hs-CRP after 2 hours following the SFA meal) and non-responders (i.e., with lower or no elevation of hs-CRP). Table 2 summarizes the basal characteristics of responders and non-responders. As shown in Table 2, the hs-CRP responders comprised 28% of the participants. In this group, 40% were men, compared with the non-responders, where the percentage of men was 26%. Weight and BMI were statistically lower in the responders than in the non-responders. A statistically significant difference was observed in the hip circumference between the responders and non-responders, 98.8±1.8 cm vs. 106.2±2.1 cm. As shown in Figure 1, the increase in triglyceride levels after 4 hours was significantly higher in the responders (141.5%) than in the non-responders (77.3%). In the non-responders, the postprandial increase in the triglyceride level stopped after 2 hours, while in the responders the triglycerides continued to rise after 2 and 4 hours.

**Table 2:** Basal characteristics of responders and non-responders, mean ± SEM

	**Responders n=15 (28%)**	**Non-responders n=39 (72%)**	**P value**
Gender	9 women (60%); 6 men (40%)	29 women (74%); 10 men (26%)	
Age (years)	39.7±4.0	44±2.2	0.5
Weight (kg)	69.5±3.0	76.2±3.1	0.02
Height (meter)	1.7±0.02	1.7±0.02	0.4
BMI (kg/m^2^)	24.8±1.0	27.1±0.9	0.02
Waist (cm)	90±3.3	93.3±2.8	0.1
Hip (cm)	98.8±1.8	106.2±2.1	0.006
Hs-CRP	2.8±0.6	4.2±1.1	0.2

Responders: at least 20% increase in the hs-CRP values 2 hours after the high SFA meal; BMI, body mass index.

xThe effects of the two meals on the metabolic markers within the treatment groups are summarized in Table 3. All the metabolic markers (insulin, glucose, HOMA-R, QUICKI and triglycerides) were affected by the time (*p*≤0.0001) and no interactions were observed. As expected, glucose levels increased significantly after both meals (*p*≤0.0001). This increase was higher after the MUFA meal than after the SFA meal (*p*=0.01), still remaining within the normal range. A statistically borderline (*p*=0.06) interaction between the meal and the time was found for the insulin concentration: following the MUFA meal, the increase in insulin values (women and men pooled) after 2 hours was slightly lower, and the decrease after 4 hours was slightly higher (128.6% and -33.8%, respectively), than following the SFA meal (154.5% and -28.8%, respectively).

**Table 3:** SFA and MUFA meals: comparison of the metabolic markers within the treatment groups, mean ± SEM

**Treating groups meals**		**Insulin (μ u/ml)**	**Glucose (mg/dl)**	HOMA-R (ins*glu/405)	**QUICKI 1/(log ins + log glu)**	**TG (mg/dl)**
SFA: Women	0h	24.7±1.8	82.6±2.1	4.9±0.4	0.31±0.01	112.3±13.4
	2h	60.3±6.7	83.9±2.7	13.4±2	0.28±0.01	191.9±22.8
	4h	43.0±4	89.9±1.4	9.0±0.9	0.29±0.01	223.2±34.6
MUFA: Women	0h	24.6±2.1	82.7±2.2	5.02±0.4	0.31±0.01	106.3±13.7
	2h	57.1±5.5	94.5±3.4	13.9±1. 8	0.27±0.01	156.9±18.2
	4h	36.0±3.2	92.8±1.7	8.2±0.9	0.29±0.01	141.8±20.3
SFA: Male	0h	24.9±2.9	90.2±7.5	5.8±1.1	0.3±0.01	147.2±24.1
	2h	70.4±13.4	97.7±9.4	18.4±4.5	0.27±0.01	255.5±33.7
	4h	48.1±7.5	96.7±5.8	12.31±2.4	0.28±0.01	340.7±62.1
MUFA: Male	0h	24.1±3.6	91.2±8.5	5.96±1.4	0.31±0.01	142.1±25
	2h	54.4±8.5	101.4±10.6	14.45±3.2	0.28±0.01	208.3±34.3
	4h	39.6±4.7	103.7±7.9	10.1±1.8	0.28±0.01	236.9±49.6
**Main effects**						
Meal		*0.07*	**0.01**	0.3	0.2	**<0.0001**
Time		**<0.0001**	**<0.0001**	**<0.0001**	**<0.0001**	**<0.0001**
0-2		**<0.0001**	**<0.0001**	**<0.0001**	**<0.0001**	**<0.0001**
2-4		**<0.0001**	0.5	**<0.0001**	**<0.0001**	**0.005**
0-4		**<0.0001**	**<0.0001**	**<0.0001**	**<0.0001**	**<0.0001**
Gender		0.6	0.1	0.3	0.5	*0.08*
**Interaction**						
Meal x Time		*0.06*	0.1	0.25	0.7	**<0.0001**
0-2						**<0.0001**
2-4						**<0.0001**
0-4						**<0.0001**
Meal x Gender		0.4	0.8	0.3	0.3	0.6
Time x Gender		0.6	0.8	0.4	0.7	0.1
Meal x Time x Gender		0.6	0.3	0.4	0.4	0.7

TG, triglycerides; HOMA, homeostasis model assessment; QUICKI, quantitative insulin-sensitivity check index; SFA, high saturated fat meal; MUFA, high monounsaturated fat meal.

Triglyceride levels were significantly affected by both the meal and the time (*p*≤0.0001 for both). The gender had a borderline effect (*p*=0.08), with higher values for the men than for the women. There was a highly significant interaction between the meal and the time: the increase in triglyceride values after 4 hours was significantly higher in both the women (98.7%) and the men (131.4%) after the SFA meal than after the MUFA meal (33.4% for the women and 66.7% for the men). The differences between hs-CRP responders and non-responders with reference to the TG level were mentioned above. No statistically significant interactions were observed with other metabolic parameters with reference to the hs-CRP in the responders.

## Discussion

An inflammatory response that follows prolonged food intake is not a novel concept [6,32-42]. This inflammatory response could contribute to the possible pathogenesis of atherothrombotic disease [43-45]. It is possible, then, that the beneficial effects of certain diets might be through their ability to attenuate the inflammatory response [46]. It is also accepted that increased long-term fat intake results in the synthesis of atherogenic lipoproteins [47], and that such dietary changes might be a potential initiator of an inflammatory response [6,34,37,39,40]. One of the goals of this work was to elucidate whether one single meal can affect postprandial elevation of inflammatory and metabolic markers in non obese apparently health subjects, thus having a pro-inflammatory. There is not much data on the effect of a single meal as a potential inflammatory initiator. Peairs et at, studied the acute effect of a single meal with different fat composition on 11 overweight and obese volunteers with no increase in inflammatory factors measured. [48] The participants in Peairs's study were supplied milk shakes of different fat composition. In our study, 54 non obese subjects were given regular popular meals, and the results showed different effect.

As effectors of the inflammatory response, inflammation-sensitive biomarkers might contribute to the appearance of insulin [11], endothelial activation [49] as well as vascular injury [50]. C-reactive protein (CRP), for instance, is not only a marker for cardiovascular risk and a predictor of coronary disease [51,52], it might also play a role in the development of atherosclerotic plaque [53,54].

We investigated whether the fat composition of a single meal, rather than its fat amount, has any effect on selected metabolic and inflammation-sensitive biomarkers which are involved in the atherosclerosis process in non obese subjects. The study supports the notion that an early postprandial inflammatory response may be related to the appearance of insulin resistance and hypertriglyceridemia. The main finding of the present study was the elevation of the hs-CRP level within 2 hours and prolonged hypertriglyceridemia within 4 hours as a result of a high saturated fat meal in apparently healthy non obese participants. ESR and WBC had different kinetics compared with hs-CRP, but it was apparent that they all increased following the SFA meal. The delineation of the interval of time from fat consumption to its expression by a biological marker that demonstrated an increased level of hs-CRP in the blood is also highly relevant to the process of macronutrient-induced inflammation and to the inflammatory response in individuals with insulin resistance [11,14].

The present experiment shows the short-term effect of macronutrients on the inflammatory response 2 and 4 hours following high SFA and high MUFA meals. It is highly possible that the effect may occur earlier. Our results showed that one meal had modest pro-inflammatory properties and the other did not, depending on the controlled macronutrient content. The main difference between the two meals in the current study was in their fatty acid composition. Unlike in the recent study of Nappo et al., there was no difference between the total amount of fat of the two meals [33].

The hs-CRP and ESR levels increased modestly but significantly statistically following the high SFA meal but not following the high MUFA meal. We showed that the hs-CRP levels were affected by the SFA meal differently in women and in men, with a higher increase in men than in women, which shows a gender-related effect of the meals. Gender differences should be taken into consideration when prescribing diet manipulations. Poppitt et al [55] showed that when giving a high fat breakfast to 18 healthy normal weight volunteers, that during the first 3 hours after the high fat meal, there were no differences in the levels of TNF alpha and IL6, as in our study, and there was no increment in the CRP level. Our study showed a moderate but significant increase in CRP levels, probably because of a difference in fat composition of the high fat meals in both studies, as well as because of a presence of responders and non-responders. However, Wood et al [56] did demonstrate in 14 obese patients with asthma that consumed a high-fat/high energy meal, that there were small increases in plasma IL-6 compared with baseline at 2, 3, and 4 hours, as well as an increase in plasma CRP at 2 hours following the meal.

In spite of the fact that the ESR was higher in women than in men, the reduction was similar in both genders. As expected, the WBCC value increased significantly after both meals [57-59], but continued to climb between 2 and 4 hours after a high SFA meal, whereas it stayed the same when measured 4 hours after a high MUFA meal. The triglyceride levels increased by 2 hours after both meals, but continued to increase only after the high SFA meal.

Since postprandial hypertriglyceridemia accelerates atherosclerotic damage by initiating inflammation and affecting the endothelium [57,60-63], it is important to define meals that can lead to a higher and more prolonged triglyceride increment and lipo-toxicity. In this study, the average triglyceride levels in the fasting state were normal, increased as expected after both meals, but continued to increase only after the high SFA meal, more profoundly in men than in women.

These findings also show the importance of measuring triglyceride levels not only following 12-14 hours of fasting as accepted nowadays, but also postprandially, in order to demonstrate their inflammatory effect. Since most people eat every few hours, it is reasonable to assume that following repeated high SFA meals they have continuous high levels of triglycerides and low-grade inflammation, with a borderline effect for gender.

In a study by Masson CJ et al [48], 50 grams of butter or sunflower margarine were added into mixed meals of 13 overweight participants; no differences in postprandial changes in TG levels between two meals were noticed. In our study, a profound difference between the effects of two meals on triglycerides level of 54 non obese participants was observed. We believe, that the large differences between different studies can be due to participants genetic variations, their number, gender and metabolic state (non obese vs. obese).

Little is known about the time course of postprandial triglyceride serum levels and their relation to the appearance of a macronutrient-induced inflammatory response. Their importance may lie in the fact that an early postprandial inflammatory response might precede a potentially hazardous transient state of insulin resistance and hypertriglyceridemia [11]. In fact, mediators of inflammation have been clearly shown to be involved in the induction of an insulin-resistance state [12,13]. A transient, macronutrient-related, microinflammatory response could explain, at least in part, the appearance of a dynamic insulin-resistant state which could be responsible for a postprandial hypertriglyceridemic response [14-23]. These data raise the possibility that the microinflammatory response could be a potential target for anti-inflammatory and antioxidant interventions as part of the efforts to lower the risk of atherosclerosis [34,40]. Since postprandial hypertriglyceridemia accelerates atherosclerotic damage by initiating inflammation and affecting the endothelium [57,60-63], it is important to define meals that can lead to a higher and more prolonged triglyceride increment.

Another important finding of this study was the fact that 28% of the cohort of apparently healthy individuals had moderately elevated hs-CRP levels 2 hours after the SFA meal. These responders differed from the rest of the participants by their anthropometric measurements, as well as by a significant higher increase in triglycerides 2 and 4 hours after the SFA meal. This severe postprandial hypertriglyceridemia reduces insulin sensitivity and causes more serious metabolic abnormalities. These findings can point to an association between genetic variations in CRP and a risk of cardiovascular disease. Other studies are needed to elucidate the genetic background of the responders [64].

We conclude that metabolic and modest inflammatory effects occur within a few hours after the ingestion of a high SFA meal in healthy subjects. Whether the speed of expression is different among individuals with diabetes mellitus awaits further investigation. Both meals were isocaloric and contained equal amounts of fat. These findings show the importance of the macronutrient composition of meals for slowing down or speeding up postprandial hypertriglyceridemia.

## Competing interests

The authors declare that they have no competing interests.

## Authors’ contributions

OR and SB have participated in the design of the study, performed the statistical analyses and drafted the paper. SB, OR, IS, conceived the study, participated in its design and coordination and helped to draft and review the manuscript. DJ and AS helped in the data organization and retrieval, English editing and final draft preparation. RT have performed the laboratory analysis on cytokines and adhesion molecules and helped in drafting the paper. All of the authors have read and approved the final manuscript.
